# Dissociable Neural Processes Underlying Risky Decisions for Self Versus Other

**DOI:** 10.3389/fnins.2013.00015

**Published:** 2013-03-20

**Authors:** Daehyun Jung, Sunhae Sul, Hackjin Kim

**Affiliations:** ^1^Laboratory of Social and Decision Neuroscience, Department of Brain and Cognitive Engineering, Korea UniversitySeoul, South Korea; ^2^Laboratory of Social and Decision Neuroscience, Wisdom Science Center, Korea UniversitySeoul, South Korea; ^3^Laboratory of Social and Decision Neuroscience, Department of Psychology, Korea UniversitySeoul, South Korea

**Keywords:** fMRI, self–other decision, amygdala, dorsomedial prefrontal cortex, risky decision, prosocial behavior, social neuroscience

## Abstract

Previous neuroimaging studies on decision making have mainly focused on decisions on behalf of oneself. Considering that people often make decisions on behalf of others, it is intriguing that there is little neurobiological evidence on how decisions for others differ from those for oneself. The present study directly compared risky decisions for self with those for another person using functional magnetic resonance imaging (fMRI). Participants were asked to perform a gambling task on behalf of themselves (decision-for-self condition) or another person (decision-for-other condition) while in the scanner. Their task was to choose between a low-risk option (i.e., win or lose 10 points) and a high-risk option (i.e., win or lose 90 points) with variable levels of winning probability. Compared with choices regarding others, those regarding oneself were more risk-averse at lower winning probabilities and more risk-seeking at higher winning probabilities, perhaps due to stronger affective process during risky decisions for oneself compared with those for other. The brain-activation pattern changed according to the target, such that reward-related regions were more active in the decision-for-self condition than in the decision-for-other condition, whereas brain regions related to the theory of mind (ToM) showed greater activation in the decision-for-other condition than in the decision-for-self condition. Parametric modulation analysis using individual decision models revealed that activation of the amygdala and the dorsomedial prefrontal cortex (DMPFC) were associated with value computations for oneself and for another, respectively, during risky financial decisions. The results of the present study suggest that decisions for oneself and for other may recruit fundamentally distinct neural processes, which can be mainly characterized as dominant affective/impulsive and cognitive/regulatory processes, respectively.

## Introduction

In daily life, we make decisions on behalf of others as often as we make decisions on behalf of ourselves: we sometimes order a lunch for a friend, choose presents for family, make decisions for a company, or buy products or stocks for customers. These other-regarding decisions, albeit not immediately targeted toward ourselves, can be critical to the establishment and maintenance of our social lives. Like decisions for oneself, decisions for others ranging from mundane to profound also involve some level of risk. Thus, it is important to understand the mental processing that drives risky decisions for others as well as those for oneself. Despite the significance of this issue, few neuroimaging studies have directly compared decisions for oneself with those for others, and only a small body of literature on the subject exists in the field of social psychology. Thus, the goal of the present study is to understand whether and how decisions (i.e., a risky decision in a gambling task) for oneself and for others differ from each other at the neural level through the use of functional magnetic resonance imaging (fMRI).

An emerging body of literature on self–other decision making has documented risky decisions in various domains, such as surrogate decisions in medicine (Hare et al., 1992; Fagerlin et al., 2001; Lipkus et al., 2001), public policy (Roszkowski and Snelbecker, 1990; Reynolds et al., 2009), career choice (Kray and Gonzalez, 1999), romantic relationships (Beisswanger et al., 2003; Wray and Stone, 2005), and financial decisions in gambling tasks (Hsee and Weber, 1997; Loewenstein et al., 2001; Stone et al., 2002; Fernandez-Duque and Wifall, 2007). Although some progress has been made, the findings of these studies have been rather inconsistent. For instance, some studies have reported that people behaved/thought in a more risk-seeking manner when they decided for another person than for themselves (Hsee and Weber, 1997; Beisswanger et al., 2003), whereas others found that people became more risk-averse in similar situations (Fernandez-Duque and Wifall, 2007).

In order to reconcile the conflicting findings listed above, recent studies have considered the potential mediating factors of these observations (Fernandez-Duque and Wifall, 2007; Stone and Allgaier, 2008). For example, Fernandez-Duque and Wifall (2007) examined actor-observer asymmetry in risky decisions and proposed that the self–other discrepancy could be mediated by differential access to experiential and rational decision making systems. They suggested that when people decide for themselves, the experiential system – which involves intuitive and emotionally based processes – might weigh more heavily on the decision making process than the rational system – which engages effortful, logical, and analytical processes (Denesraj and Epstein, 1994). This could be the case because actors who make decisions for themselves are more likely to be influenced by their own affective reactions to the consequent rewards and punishments. Similarly, Hsee and Weber (1997) showed that the level of description of the other person accessible to participants mattered in the self–other decision making discrepancy. In their study, participants predicted that others would be more risk-seeking than they would be in terms of financial decisions when the other person for whom the decision was made was described in anonymous and abstract terms. However, the self–other difference diminished when the other person for whom the decision was made was described vividly and in concrete terms. The authors suggested that a vivid description of the other person made the decisions for self and for other commensurate by eliciting strong affective reactions in subjects. To explain the findings, they proposed a “risk-as-feelings hypothesis,” which maintains that people rely on affective evaluations when making decisions for themselves in risky situations (Hsee and Weber, 1997; Loewenstein et al., 2001).

The idea that risky decisions for oneself are mainly affected by emotional reactions has been supported by a large body of neuroscience literature. Most relevant is the finding that the amygdala, a key structure for emotional processing during decision making (Morrison and Salzman, 2010), plays a critical role in risky decision making (Bechara et al., 1999; Hsu et al., 2005; De Martino et al., 2006, 2010; Brand et al., 2007; Ghods-Sharifi et al., 2009; Smith et al., 2009). For instance, De Martino et al. (2006, 2010) studied the neural correlates of the framing effect, whereby people become more risk-averse in a gain frame (i.e., when gains are made salient) than in a loss frame (i.e., when losses are made salient). This effect is a representative example of emotionally driven decision making in risky situations and was strongly associated with activity in the amygdala (De Martino et al., 2006); further, the effect was significantly diminished in patients with amygdalar damage (De Martino et al., 2010).

The amygdala forms extensive anatomical and functional connections with the dorsomedial prefrontal cortex [DMPFC, which includes Brodmann areas (BAs) 9, 32, 33, and part of the medial prefrontal cortex (MPFC); Etkin et al., 2011] that show developmental progress (Hung et al., 2011). The amygdala’s affective reactions seem to be regulated *via* these connections (Banks et al., 2007; Kim et al., 2011). Although relatively little is known about the role of these amygdala–DMPFC connections in risky decisions (Cohen et al., 2005), the DMPFC itself is also known as a key structure for decision making in risky situations. For example, Wu et al. (2011) found that activation of the MPFC, including both dorsal and ventral regions, quantitatively reflected the subjective value of monetary outcomes combined with probability information about lottery tasks. Another study showed that the DMPFC was specifically responsive to risk-related information (Xue et al., 2009). Similarly, many previous studies using the Iowa Gambling Task (IGT) have shown that risky decision making is associated with increased activation of the DMPFC (Bolla et al., 2003; Fukui et al., 2005; Tanabe et al., 2007). Further, the DMPFC plays an important role in emotional regulation during affective decision making (Banks et al., 2007), effort-based decision making (Rudebeck et al., 2006; Floresco and Ghods-Sharifi, 2007; Croxson et al., 2009), and perspective taking during other-regarding processes (St. Jacques et al., 2010). In sum, while the amygdala is responsible for affective reactions in risky decisions, the DMPFC seems to control cognitive processes, such as weighing the probabilities and reward values of different options and regulating emotion.

As reviewed above, evidence from the social psychology literature implies the existence of distinctive neural circuitry subserving decisions on behalf of others as opposed to those made for oneself, and unveiling this difference would greatly advance the current theoretical account of prosocial decisions. In line with this idea, a recent study showed that activity in the ventromedial prefrontal cortex (VMPFC) was modulated by activity in the inferior parietal lobule (IPL) – a brain region close to the temporoparietal junction (TPJ) that is involved in mentalization (Saxe and Powell, 2006) – when people made product purchase decisions for others, whereas no such modulation effect of TPJ was found when people made the same decisions for themselves (Janowski et al., 2012).

The present study aimed to examine the difference between decisions made for oneself and those made for another in a risky situation by using a gambling task paradigm with systematically variable winning probability. On the bases of previous findings, we predicted that affective processes would have stronger weight in decisions made for oneself than for other. Thus, we hypothesized that considering a risky choice on behalf of another may employ the brain regions involved in cognitive/rational processes (e.g., the prefrontal cortex) more than those associated with affective/experiential processes (e.g., the amygdala), whereas the opposite may be true when the risky decision is made for oneself.

## Materials and Methods

### Participants

Twenty-three undergraduate students in South Korea [12 women; mean age (SD) = 23.32 (2.59)] participated voluntarily and were compensated an average of KRW 30000 (∼USD 25) for about 1 h of participation. Any potential health risks were carefully screened *via* a self-report questionnaire, and informed consent was obtained from all participants. All participants were right-handed and reported having no chronic mental illness. Three participants were excluded from analysis because they fell asleep inside the scanner. The experimental procedures were approved by the Institutional Review Board of Korea University.

### Task and procedures

**Figure 1:**
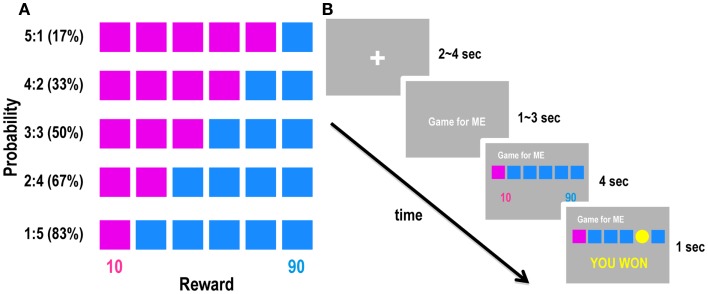
**Schematic diagram of the experimental design**. **(A)** Five experimental conditions with variable probabilities and fixed outcomes. Percentages in parentheses indicate winning probabilities of the high-risk option (blue). **(B)** A schematic diagram of the experimental design. Each trial began with a cue indicating whether the decision is for self or for other, followed by a gambling task. Participants were then asked to choose one of two colors (pink or blue) by clicking the left or right mouse button.

Participants performed a gambling task inside the MRI scanner. We adopted the “modified risk task” developed by Knoch et al. (2006), in which participants were asked to choose between two options: one with lower risk (i.e., win or lose 10 points) and another with higher risk (i.e., win or lose 90 points). The winning probability of each option was 17–83% (the probabilities used were 17, 33, 50, 67, and 83%). In each trial, participants were presented with six boxes distinguished by pink and blue colors, and they were asked to choose either pink or blue. The colors of the boxes indicated the numbers of points that the participants could win or lose: 10 for pink and 90 for blue (Figure 1A). Participants were told that a yellow coin had an equal chance to appear in any of the boxes and they would gain points if the coin was contained in one of the boxes with the chosen color and that they would lose the same number of points if the coin appeared in an opposite-colored box. For example, if they chose pink and there was a coin in one of the pink boxes, they won 10 points, but if there was no coin in the chosen color, they lost 10 points. Likewise, if they chose blue, they won or lost 90 points if there was or was not a coin among the blue boxes, respectively. Thus, pink were the low-risk and blue were the high-risk options. The ratio of pink and blue boxes determined the winning probability of each option; for example, five pink boxes and one blue box meant that the winning probabilities were 83% for pink and 17% for blue. In each trial, participants had to make a decision on behalf of either themselves (decision-for-self condition) or another person (decision-for-other condition), according to a cue presented prior to the task. The structure of a single trial is shown in Figure 1B. First, the cue indicating the decision condition (decision-for-self or decision-for-other) was presented for 1–3 s, followed by the risk task. After the six boxes were presented, the participants chose between pink and blue by pressing the left or right button of an MR-compatible mouse. They were asked to respond carefully but as quickly as possible. Each participant performed 120 total trials (60 in each of the decision-for-self and decision-for-other conditions), which were divided into three sessions. Each session consisted of 40 trials, with the same number of trials for each condition. The order in which the different types of trials were presented was determined pseudorandomly. Earned points were accumulated separately for each condition. Participants were told that the points accumulated in all trials would be converted into real money. We informed participants that they would be endowed with a base payment of 30,000 KRW; we also informed them that 25000–35000 KRW was an approximate range of final compensation. Participants were kept blind to the exact ratio between points and money, because we did not want them to focus on calculating the exact amounts of money earned by themselves or others. Subjects were also told that their task performance in the decision-for-other condition would determine extra earnings of another person who was randomly selected among the participants of the same experiment. Participants understood that the transfer would be completely anonymous so that neither the participants themselves nor their counterparts would know each other’s identities. They also knew that their decisions for others would not affect their own profits, because the point totals for self and other were calculated separately, and the tasks were performed individually. In both conditions, participants started with 100 points; 4 s after the participants made a decision, the result of the decision (i.e., win or lose) was presented on the feedback screen (Figure 1B).

All instructions were given outside the scanner, and each participant performed 10 practice trials before entering the scanner to learn the task rules. After the completion of the task, the points earned during the decision-for-self condition were converted into money and added to the subject’s base payment (KRW 30000), and the points earned during the decision-for-other condition were actually transferred to another participant for whom the participant made the decisions. Participants’ final earnings varied 25000–35000 KRW, depending on their own performance and that of the other randomly matched participant.

### Neuroimaging procedures

#### fMRI data acquisition

We acquired data using an ISOL Forte 3T system with a standard birdcage coil in the Brain Science Research Center at the Korea Advanced Institute of Science and Technology. T2*-weighted functional images were obtained using gradient–echo echo-planar pulse sequences (TR = 2000 ms; TE = 30 ms; FA = 80°; FOV = 240 mm; 64 × 64 matrix; 24 slices; voxel size = 3.75 mm × 3.75 mm × 4.0 mm). The stimuli were presented through an MR-compatible LCD monitor mounted on a head coil (refresh rate: 60 Hz; display resolution: 640 × 480 pixels; viewing angle: 30°). Each functional run lasted 480–600 s, including the first five TRs, which were discarded later due to unstable magnetization.

#### fMRI data analyses

Neuroimaging data were preprocessed and analyzed using Statistical Parametric Mapping 8 (SPM8; the Wellcome Trust Centre for Neuroimaging, University College London, UK). After timing correction for interleaved slice acquisition, correction for head motion was performed through realignment of all functional images to those of the first scan, and then a mean brain image was created for each participant. The realigned images were normalized to the Montreal Neurological Institute (MNI) echo-planar imaging (EPI) template, resampled at a voxel resolution of 2 mm × 2 mm × 2 mm, and spatially smoothed with an 8 mm Gaussian filter. Anatomical localization was performed in SPM8 by reference to a T1 template image, and then the preprocessed functional images were analyzed using the general linear model (GLM; Friston et al., 1995). Regressors for the events of choice and outcome delivery were convolved with a canonical hemodynamic response function. Motion vectors obtained from the realignment process were included as regressors in the GLM in order to reduce noise. The resulting statistical parametric maps were first thresholded stringently (significance level: FWE < 0.05, corrected for multiple comparisons; cluster size threshold >5 voxels), but no activation cluster survived this threshold.

To verify *a priori* hypotheses regarding several regions of interest (ROI), we used the small-volume correction (SVC) method for multiple comparisons (*p* < 0.05) in SPM8. We expected that the ventral striatum (VS; left: *x* = −10, *y* = 6, *z* = −14; right: *x* = 8, *y* = 6, *z* = −10), the ventral tegmental area (VTA; *x* = −4, *y* = −14, *z* = −20), the anterior cingulate cortex (ACC; *x* = 4, *y* = 24, *z* = 40), and the insula (*x* = −34, *y* = 20, *z* = −4) would be involved in reward anticipation and feedback during risky choice in the self–other contrast (Ernst et al., 2004) and that the TPJ (left: *x* = −48, *y* = −57, *z* = 25; right: *x* = 53, *y* = −54, *z* = 17), the posterior cingulate cortex (PCC; *x* = 2, *y* = −60, *z* = 27), and the MPFC (*x* = 1, *y* = 63, *z* = 2) – which are known to be related to theory of mind (ToM) functions (Saxe and Powell, 2006) – would be involved in the same function in the other–self contrast. In addition, we expected the amygdala (*x* = 22, *y* = −4, *z* = −18; Smith et al., 2009) and the DMPFC (*x* = −8, *y* = 36, *z* = 30; Wu et al., 2011) to encode the value of the risky option. The search volumes for SVC were restricted to spheres with radii of 15 mm and center coordinates obtained from corresponding studies. Additionally, we defined the ROIs in both hemispheres by mirroring the coordinates obtained in previous studies. To reduce the risk of false negatives and completely overview the clusters at which activation occurred, we also applied a less-stringent significance level (*p* < 0.001, uncorrected; cluster size threshold ≥5 voxels); a table with a list of activation clusters is included in the Supplementary Material.

Brodmann areas and brain regions were identified in Talairach space (Talairach and Tornoux, 1988) after converting the MNI coordinates to Talairach ones using non-linear transformation (Lancaster et al., 2007).

#### Contrasting decision-for-self versus decision-for-other

In order to explore which brain regions were more highly activated by the decision-for-self task than by the decision-for-other task or vice versa, we estimated whole-brain contrast maps from the periods when participants watched the six boxes and received reward information during both tasks. The single-subject whole-brain GLMs included the following regressors: (1) decision events at the time of task onset, when participants viewed the stimuli for 10 types of trials (i.e., five levels of probability in both the decision-for-self and decision-for-other conditions) and made decisions, (2) button-pressing events, (3) feedback events (at feedback onset, when participants watched two types of outcomes: those for self and other), and (4) motion parameters. The self–other and other–self contrasts were defined for all probability conditions.

#### Parametric modulation analysis based on individual decision models

We conducted parametric modulation analysis to determine which brain regions had activation levels that correlated with the decision value that each participant placed on the risky choice. Each participant made risky choices for self and other with varying probabilities of a favorable outcome; we calculated the decision value using optimal sigmoid functions fitted to the participant’s probability (0–1) of choosing the high-risk option over the low-risk option as a function of the probability of winning. The parameters of the estimated models were calculated by using the least-squares method for each participant (see the equation below).

$$f(x_{i})=\frac{1}{e^{a(b-x_{i})}+1}$$

In the equation above, the variable *x* is the winning probability of the high-risk option, and *f*(*x_i_*) is the probability of a risky choice on trial *i*. The parameter *a* indicates the slope of the sigmoid function that reflects how drastically the probability of risky choice changes according to the level of winning probability, and *b* denotes an offset criterion for the winning probability of the high-risk option when the participant is expected to choose the risky option with 50% probability. We calculated the parameters separately for the decision-for-self and decision-for-other conditions. We generated separate single-subject GLMs for parametric modulation analysis, which included the following regressors: (1) decision events when a new configuration of colored boxes is displayed on the computer screen, along with individually estimated decision values [i.e., *f*(*x_i_*)] as parameters for the decision-for-self and decision-for-other conditions; (2) button-pressing events; (3) feedback events at the time of feedback onset when participants watched two types of outcomes (i.e., those for self and other); and (4) motion parameters.

#### Psychophysiological interaction analysis

We conducted psychophysiological interaction (PPI) analysis (Friston et al., 1997) to examine the functional connectivity between the brain regions identified from the contrast analyses. Specifically, we searched for brain regions whose activity showed differential patterns of correlations with that of a source region as a function of experimental condition (i.e., the decision-for-self and decision-for-other conditions). We used the right TPJ (rTPJ) as the source region, because it is the representative area that reflects both perspective taking (Castelli et al., 2000; Saxe and Wexler, 2005; Decety and Lamm, 2007) and other-regarding behavior (Morishima et al., 2012), and because we focused on examining how the brain regions activated during decision-for-other communicated with other areas. We extracted time-series data from the peak voxel of a cluster found in rTPJ for each participant and then generated PPI regressors, that is, the time-course of activity in the seed region modulated by two levels of the psychological variable (i.e., decision-for-other versus decision-for-self). We then estimated single-subject whole-brain GLMs with the following regressors: (1) time-course of activity in the seed region (rTPJ activity), (2) psychological contrast (other–self contrast weight), (3) interaction term (rTPJ activity × other–self contrast weight), and (4) motion parameters.

## Results

### Behavioral results

**Figure 2:**
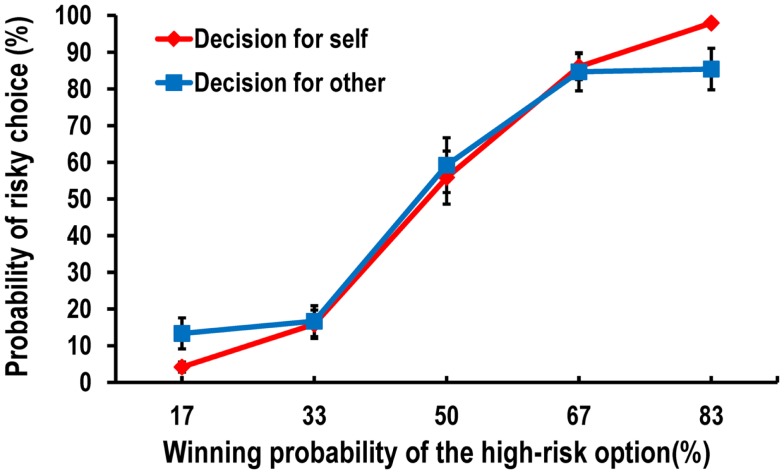
**Behavioral data showing the probability of risky choice as a function of the probability of a more favorable outcome**.

We first calculated the ratio of risky choices to the total number of trials in each condition for each participant. Then, we conducted a 2 (conditions: decision-for-self and decision-for-other) × 5 (winning probabilities of the high-risk option: 17, 33, 50, 67, and 83%) repeated-measures ANOVA on the probability of choosing the high-risk option (i.e., blue) over the low-risk option (i.e., pink). Because Mauchly’s test indicated that the assumption of sphericity had been violated (χ^2^= 34.070, *p* < 0.05), we used a multivariate test, which revealed a significant two-way interaction effect, *F*(4, 16) = 3.150, *p* < 0.05. As shown in Figure 2, the difference between the frequencies of high-risk decisions for self and for other varied according to the probability of winning. Participants were more likely to make risk-seeking decisions in the decision-for-self condition than in the decision-for-other condition when the winning probability of the high-risk option was higher, while the reverse was true when it was lower.

To investigate this interaction further, we conducted *post hoc* pairwise *t*-tests on the differences in the risky choice ratio between the decision-for-self and decision-for-other conditions at each level of wining probability of the higher risk option. We found a significant difference between the conditions at 83%, *t*(22) = 2.319, *p* < 0.05, and a marginally significant difference at 17%, *t*(22) = −2.01, *p* = 0.059 (Figure 2), although none of the tests survived Bonferroni correction.

### Neuroimaging results

#### Decision-for-self versus decision-for-other during the decision event

**Figure 3:**
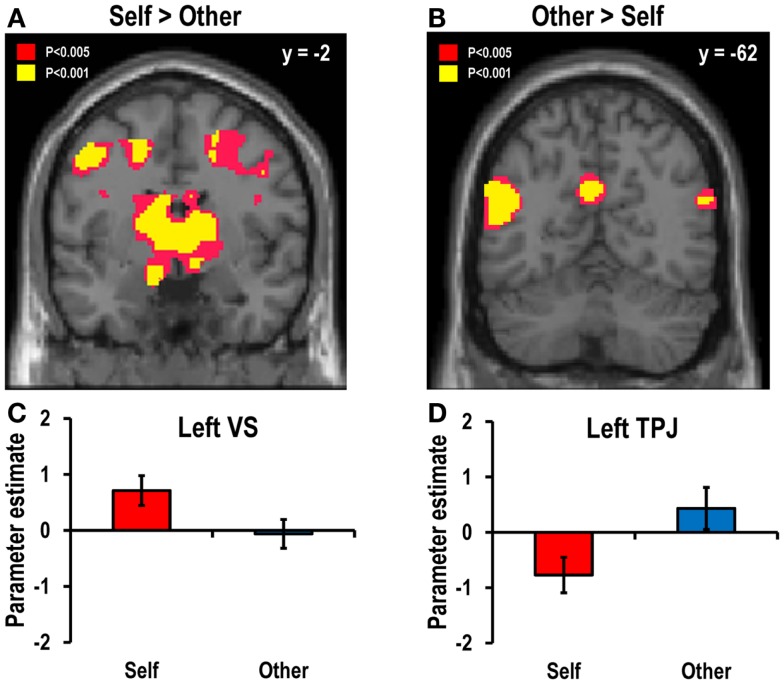
**Main contrast maps between self and other conditions**. Areas showing greater activity during decision events **(A)** in the decision-for-self condition than in the decision-for-other condition and **(B)** in the decision-for-other condition than in the decision-for-self condition. The statistical threshold for the images was set at *p* < 0.005 (uncorrected). The bar graphs in the lower panel show the beta coefficients (averaged across all probabilities) of **(C)** the left VS (*x* = −12, *y* = −2, *z* = −14; *Z* = 4.19, *p* < 0.05, SVC FWE-corrected) for the self–other contrast and **(D)** the left TPJ (*x* = −50, *y* = −62, *z* = 16; *Z* = 4.34, *p* < 0.05, SVC FWE-corrected) for the other–self contrast.

To compare the brain regions associated with decisions for oneself with those associated with decisions for another, the self–other and other–self contrasts at the time of decision (i.e., task onset time) were estimated. The self–other contrast revealed greater activation in the decision-for-self condition than in the decision-for-other condition in various regions, including the bilateral VS (Figures 3A,C; left: *x* = −12, *y* = −2, *z* = −14; right: *x* = 18, *y* = 12, *z* = −16), the VTA (*x* = 6, *y* = −24, *z* = −18), the ACC (*x* = 8, *y* = 36, *z* = 34), and the right insula (*x* = 34, *y* = 24, *z* = −12; all findings thresholded at *p* < 0.05, SVC FWE-corrected unless otherwise stated). The other–self contrast showed that the bilateral TPJ (left: *x* = −50, *y* = −62, *z* = 16; right: *x* = 58, *y* = −66, *z* = 24) and the PCC (*x* = −6, *y* = −58, *z* = 30) were more active in the decision-for-other condition than in the decision-for-self condition (Figures 3B,D).

#### Neural responses to monetary outcomes for self versus other

The self–other contrast at the time of the monetary outcome events revealed preferential activation of the right insula (*x* = 32, *y* = 18, *z* = −16) in the decision-for-self condition. The other–self contrast revealed the opposite pattern in the bilateral TPJ (left: *x* = −48, *y* = −62, *z* = 24; right: *x* = 48, *y* = −58, *z* = 22) and the PCC (*x* = 2, *y* = −64, *z* = 38), which are similar to the areas of activation observed at the time of decision events (Figure S1 in Supplementary Material).

#### Parametric modulation analysis using individual decision models

The present study mainly aimed to examine the distinctive neural structures involved in the computation of values of choices and the prediction of risky choices on behalf of both oneself and others. With this in mind, we conducted parametric modulation analysis using the participants’ individual decision models. Model parameters were generated by fitting sigmoid functions to the probabilities of choosing the high-risk option over the low-risk option. Eighteen participants were subjected to the analysis, excluding two participants whose behavioral data fit poorly to sigmoid functions because of their atypical decisions (e.g., risky choices regardless of probability). The individual decision models were estimated for the decision-for-self and decision-for-other conditions separately.

The analyses revealed that the chance of making a risky choice for self was positively correlated with activation of the right anterior amygdala (*x* = 16, *y* = 6, *z* = −16), whereas the chance of making a risky choice for other was positively correlated with activation of the left DMPFC (*x* = −14, *y* = 34, *z* = 32). Activation was not negatively correlated with the chance of making a risky decision-for-self or for other in any brain region.

**Figure 4:**
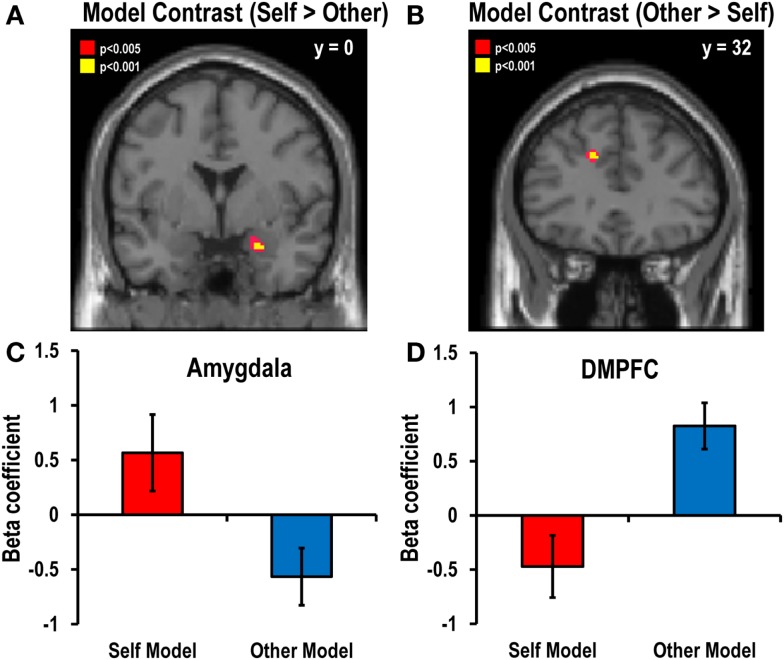
**Main contrast maps between self and other decision models**. Significant correlations with the value parameters of risky choice estimated by fitting sigmoid functions to actual decisions were found in **(A)** the right amygdala (*x* = 24, *y* = 0, *z* = − 22; *Z* = 3.85, *p* < 0.05, SVC FWE-corrected) for the self versus other contrast and **(B)** the left DMPFC (*x* = − 14, *y* = 32, *z* = 32; *Z* = 3.94, *p* < 0.05, SVC FWE-corrected) for the other versus self contrast. The statistical threshold for the images was set at *p* < 0.005 (uncorrected). The bar graphs in the lower panel show the beta coefficients of the **(C)** amygdala for the self versus other contrast and **(D)** the DMPFC for the other versus self contrast.

To investigate which brain regions drive the differences between the models for self and other, we calculated the contrast between the value computation models for self and other *via* parametric modulation analysis. The self–other contrast showed that activation in the right amygdala (*x* = 24, *y* = 0, *z* = −22) was closer to that predicted by the value computation model for self than that for other, while activation in the left DMPFC (*x* = −14, *y* = 32, *z* = 32) showed a stronger association with the decision model for other than for self (Figure 4).

#### Parametric modulation analysis using expected value and outcome

We conducted additional parametric modulation analysis to examine prediction error (PE)-related neural activity at the time of the feedback events. The PE parameters were calculated by subtracting the expected values (EVs) from the monetary outcomes (−10, 10, −90, or 90 points) separately for self and other conditions. The EV of the low-risk option (i.e., choosing the pink box) was calculated by adding the respective EVs for gain (i.e., winning probability of the low-risk option × points gained for winning) and loss (i.e., winning probability of high-risk option × points lost for losing); the EV of the high-risk option was calculated in an analogous manner. This analysis revealed that in the decision-for-self condition, activity in the ACC (*x* = 8, *y* = 32, *z* = 26) was correlated negatively with PE, whereas activation was not significantly correlated with PE in any brain area in the decision-for-other condition (Figure S2 in Supplementary Material).

#### Psychophysiological interaction analysis

**Figure 5:**
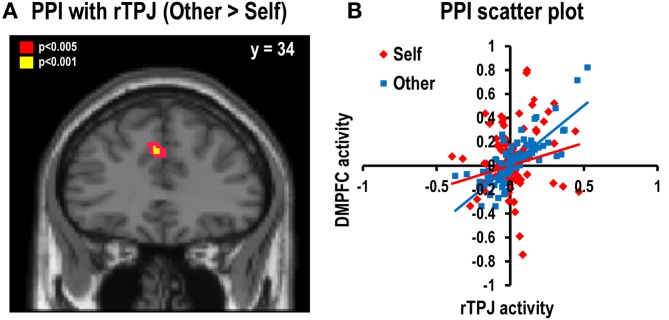
**Psychophysiological Interaction (PPI) analysis**. **(A)** Stronger functional connectivity with rTPJ was found in the left DMPFC during decisions for another than for oneself (*x* = −4, *y* = 34, *z* = 34; *Z* = 3.22, *p* < 0.001, uncorrected). The statistical threshold for the images was set at *p* < 0.005 (uncorrected). **(B)** The scatter plot representing a single-subject’s data. It shows a stronger positive correlation between rTPJ and DMPFC during the decision-for-other condition than the decision-for-self condition.

We assessed the functional connectivity between brain regions during the decision-for-self and decision-for-other conditions using PPI analysis. We identified the brain regions in which correlations between their activity levels and those of rTPJ were modulated by psychological condition (decision-for-self versus decision-for-other). The results revealed that rTPJ showed stronger positive connectivity with the left DMPFC (*x* = −4, *y* = 34, *z* = 34) in the decision-for-other condition than the decision-for-self condition (*p* < 0.001, uncorrected; Figures 5A,B). The coordinates of the DMPFC reported here are immediately adjacent to those reported from the decision model for the other–self contrast (Figure S3 in Supplementary Material).

Considering the widespread problem of non-independence error in neuroimaging research (Kriegeskorte et al., 2009), we were concerned about whether the present PPI findings in the DMPFC were independent of seed-point selection. We did not observe elevated DMPFC activity in the other–self contrast, even at a low statistical-significance threshold (*p* < 0.05, uncorrected), and careful examinations of individual subjects’ PPI GLM models revealed no evidence of a significant correlation between the TPJ–time-course regressor and the psychological variable regressor. Therefore, it seems more plausible that the variability in TPJ activity not accounted for by the regressor for the other–self contrast contributed significantly to the PPI-related activity in the DMPFC observed in the present study. This argument is further supported by the relationship between the TPJ and DMPFC activities, as exemplified by a scatter plot of a representative individual in Figure 5B. In addition, we performed cross-validation analysis using a leave-one-subject-out method (Esterman et al., 2010), in which single-subjects are iteratively left out of the first-stage group analysis that localizes the TPJ. This analysis confirmed the original results, although the size of the cluster in the DMPFC became slightly smaller (*x* = −4, *y* = 34, *z* = 34; *p* < 0.001, uncorrected; Figure S4 in Supplementary Material). In sum, although potential bias due to non-independent use of the data cannot be completely excluded, we believe the possibility that it occurred is minimal.

### Additional behavioral experiment

In order to explain the behavioral results, which were less distinguishable than the neural data in terms of self–other differences, we conducted an additional behavioral experiment in which we examined whether individual differences in prosociality explain the reduction in self–other behavioral discrepancies. When we interviewed the participants about how they felt during the task, some said that their decisions made for another person felt the same as those made for themselves, whereas others said that they could clearly distinguish between the two conditions in terms of feelings. Thus, we hypothesized that individual differences in prosociality (i.e., the ability or disposition to regard another person’s benefit as being as important as one’s own) would affect the degree of self–other discrepancy in risky decision making.

**Figure 6:**
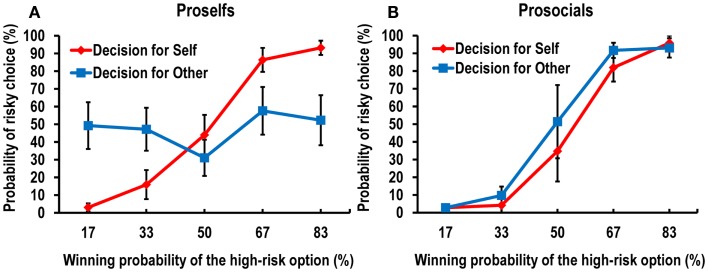
**Data from the additional behavioral study showed distinct behavioral response patterns between (A) proself participants and (B) prosocial participants**.

Nineteen participants performed the same risk tasks as we used in the main experiment. The selection of high-risk options during the task increased linearly as a function of the probability of winning; this replicated the findings of the main experiment. The statistical-analysis procedures were the same as those used for the behavioral data in the main experiment. The interaction between conditions and winning probabilities was significant, *F*(4, 15) = 3.099, *p* < 0.05. To investigate the modulatory role of individual variability, we measured each individual’s prosocial tendency with the Triple Dominance Measure (TDM) task (see Supplementary Material for details), which was adopted from a previous study (Haruno and Frith, 2010). After removing two participants who made inconsistent choices, which prevented clear categorization, we categorized the participants into three groups: prosocials (*n* = 6), individualists (*n* = 10), and competitors (*n* = 1). We then combined individualists and competitors into the proself group, following two previous related studies (Van Lange and Liebrand, 1989; Sattler and Kerr, 1991). Figure 6 shows a greater self–other distinction in the probability of risky choice for the proselfs (Figure 6A) compared with that for the prosocials (Figure 6B), although no significant three-way interaction was observed among group (proselfs versus prosocials), condition, and winning probability using a multivariate mixed ANOVA, *F*(4, 12) = 1.252, *p* = 0.341. However, the three-way interaction was significant when a mixed ANOVA was applied after Greenhouse–Geisser correction, *F*(2.216, 33.245) = 3.724, *p* < 0.05.

Additionally, we performed a correlation analysis between self–other indices (generated by squaring the difference in probability of making a risky choice between the self and other conditions) and individuals’ TDM scores (obtained by counting the number of prosocial choices across eight sets of decision trials). We found a negative relationship between prosocial tendency and self–other indices (Pearson correlation coefficient *r* = −0.523, *p* < 0.05), indicating that more prosocial participants showed smaller differences in risky choice between the self and other conditions.

## Discussion

The present study investigated the differences between the neural correlates of risky decision making on behalf of oneself and that on behalf of others *via* fMRI. The behavioral results showed that participants were more sensitive to risk-related information (i.e., probability of winning) in the decision-for-self condition than the decision-for-other condition. When participants decided for themselves, they became more risk-averse and risk-seeking when the winning probability of the high-risk option was lower and higher, respectively. This tendency became weaker when people decided for others; this might suggest diminished affective responses to the risky situation in this condition. We sought to test this possibility by contrasting the neural correlates of risky decision making for oneself with those for others. The brain-activation pattern changed according to the target of the decision, such that reward-related regions were more active in the decision-for-self condition than in the decision-for-other condition, whereas regions related to the ToM showed the opposite association. Parametric modulation analysis describing each individual’s decision model revealed that the amygdala and DMPFC were involved in computing decision values targeting self and other, respectively. These findings indicate that fundamentally distinct neural processes subserve value computations when making risky choices for oneself versus for other people.

### Self–other differences in risky decision making

Participants were more likely to vary their choices according to winning probability in the decision-for-self condition than in the decision-for-other condition. More specifically, participants made risk-seeking and risk-averse choices when the winning probability of the risky option was high and low, respectively. This may indicate greater involvement of emotional processes in biasing risky choices for self versus other. The fact that this pattern was not observed in the decision-for-other condition may indicate weak emotional intrusion or effective cognitive regulation while making choices for other. Moreover, as is the case for other types of other-regarding behavior, risky decisions for others may also require the ability to understand others’ minds. Indeed, we found in the additional behavioral experiment that the self–other difference in risk-seeking choices was affected by subjects’ levels of prosociality. That is, prosocial participants made decisions for themselves and for others in the same manner, whereas proself participants made the two types of decisions in distinguishable ways. This result hints that the ToM function may contribute to risky decision making for others, given that prosocial orientation is tightly related to perspective taking and mentalization (Underwood and Moore, 1982). In addition, the amount of effort expended deciding for another person could be another determinant of individual differences in decision making regarding self versus other. In our additional experiment, proself participants were less sensitive to probability information that is critical for successful decisions, when making choices for others than for themselves. This suggests that people who are indifferent to others’ benefit put less effort into decisions for others than those for themselves. In other words, making a decision for another person would be a painstaking task to someone who acts in the best interests of others (i.e., a prosocialist), because he/she would feel the need to reduce the fundamental self–other difference.

Overall, decision making on behalf of others seems to be a demanding process that entails expending more effort and cognitive resources than making decisions for oneself: it requires different psychological and physiological mechanisms and is more difficult. When people make decisions on behalf of others, cognitive processes are weighted more heavily than affective processes are (Fernandez-Duque and Wifall, 2007), and subjects tend to make such decisions in norm-based ways, such that they consider what they think is “right” rather than what they “feel like doing” (Stone and Allgaier, 2008). Subjects making decisions on behalf of others also seem to value their reputations (i.e., the impressions they convey to the people for whom they make the decisions; Jonas et al., 2005). At the same time, other-regarding behavior might require self-regulatory processes to deal with the conflict between selfish and prosocial motivations: subjects feel a need to regulate their emotional reactions and inhibit their selfish impulses to minimize cost, but if they do nothing, they neglect the other person’s interests (DeWall et al., 2008). Thus, it would be reasonable to think that the self–other discrepancy in risky decision making observed in the present study reflects the different types of psychological processes (i.e., affective versus cognitive processes) associated with making decisions on behalf of oneself versus others, respectively. Further, the self–other difference in the amount of cognitive resources required during the risky decision task might have resulted in behavioral differences.

### Brain regions associated with decisions for self and for other

One of the main findings of the present study is that people seem to use different modes of decision making when they decide for themselves and for others; this is particularly emphasized by the neuroimaging results. In the decision-for-self condition, the VS, caudate, VTA, insula, and ACC were more active than in the decision-for-other condition. Given the large number of previous studies that reported strong associations between these regions and both reward processing (Breiter et al., 2001; Knutson et al., 2001; Baxter and Murray, 2002; Ernst et al., 2004; Yacubian et al., 2006; Carter et al., 2009; Ghods-Sharifi et al., 2009; Smith et al., 2009) and risk processing (Kuhnen and Knutson, 2005; Preuschoff et al., 2006), people might be more sensitive to reward and perceived risk when they make decisions for their own profit than for that of others. On the other hand, the TPJ, PCC, and MPFC showed greater activation in the decision-for-other condition than in the decision-for-self condition. Given that these regions are regarded as parts of the ToM network, which is central to understanding others’ intentions through mentalization and perspective taking (Fletcher et al., 1995; Gallagher et al., 2000; Walter et al., 2004; Saxe and Wexler, 2005; Amodio and Frith, 2006; Frith and Frith, 2006; Saxe and Powell, 2006), it seems that people might activate their ToM systems in order to take another’s perspective and thus perform the risky choice task for another’s benefit. Supporting this idea, a recent study (Janowski et al., 2012) found that VMPFC activity during decision making for others – but not for oneself – was modulated by TPJ, one of the important brain regions involved in mentalization. These differences between decision making for oneself and for others may lead to self–other distinctions in the value computation and decision processes, which are discussed below.

### Neural correlates of value computation in risky decisions for the self versus for others

The most noteworthy finding of the present study is revealed by the contrast between the decision models for self-targeted versus for other-targeted decisions. The parametric modulation analysis of each individual’s decision models elucidated the distinct neural correlates of value computation for self and for other in risky decision making, revealing negative coupling between activations of the amygdala and the DMPFC whose magnitudes depended on the target of the decision. Activations in the amygdala and DMPFC were associated with value computation in the modified risk task, replicating the results of previous studies (Ghods-Sharifi et al., 2009; Smith et al., 2009; Morrison and Salzman, 2010). Importantly, the direct contrast between self-regarding and other-regarding decision making in terms of the computed value of the risky option revealed that the amygdala was more strongly associated with the value computation for self than that for other; this result is in line with the “risk-as-feelings hypothesis,” which proposes that affective responses play a relatively greater role in risky decision making for oneself than for others (Loewenstein et al., 2001). On the other hand, the DMPFC was more engaged in the value computations regarding decisions for other than those for self; this result supported our prediction that cognitive processes might outweigh affective processes in risky decision making for others.

As we reasoned above, decision making for another without regard to one’s own benefit could require effort and additional cognitive resources. In this respect, recent evidence on the role of the ACC – which is immediately adjacent to the DMPFC – in effort-based decision making might provide an interesting explanation for our findings. For example, severing the connection between the amygdala and the ACC impaired rats’ decision making abilities, such that they no longer chose a high-reward option that required more effort than the corresponding low-reward option (Floresco and Ghods-Sharifi, 2007). Studies in both animals and humans have shown that the ACC is sensitive to the amount of effort exerted during decision making and shows increased activation during increased effort to earn larger rewards in both animals and humans (Rudebeck et al., 2006; Croxson et al., 2009). Thus, it seems plausible that the stronger association between DMPFC activation and the value computation in the decision-for-other condition than in the decision-for-self condition may reflect the fact that people tend to expend greater amounts of effort during risky decision making for others than for themselves. In addition, the regulatory function of the DMPFC over amygdalar activity may play a role in creating the self–other distinction between the neural correlates of value computation. The DMPFC forms strong connections with the amygdala (Roy et al., 2009; Salzman and Fusi, 2010; Etkin et al., 2011; Hung et al., 2011; Robinson et al., 2011) and regulates its emotional reactions (Banks et al., 2007). Indeed, decision making for others requires self-regulatory processes in order to deal with the conflict between selfishness and prosociality (DeWall et al., 2008). The role of the DMPFC as part of the ToM network (Fletcher et al., 1995; Gallagher et al., 2000; Walter et al., 2004; Amodio and Frith, 2006; Frith and Frith, 2006) provides another possible explanation for the self–other distinction in the neural correlates of value computation, especially in relation to perspective taking, mentalizing, and inferring others’ intentions (St. Jacques et al., 2010). Consistent with this idea, a recent study showed that DMPFC activity during the judgment of others’ opinions (Waytz et al., 2012) or during the observation of others’ distress (Masten et al., 2011) predicted subsequent prosocial behavior. Thus, consideration of risky options for others may require inference of their mental states, which then in turn recruits the ToM network, including the DMPFC.

Consistent with the previous ToM literature, activity in the rTPJ – a major area for mentalization (Castelli et al., 2000; Saxe and Wexler, 2005; Decety and Lamm, 2007) – was greater during the decision-for-other than the decision-for-self condition in the present study. This region also showed heightened functional connectivity with DMPFC in the other–self contrast; the activity of DMPFC increased as a function of the computed values of risky option during choices for other more than during choices for self. The findings make it tempting to speculate that rTPJ may send a signal to DMPFC and contribute to its control of amygdalar activity when considering choices for others, enabling us to choose options with diminished emotional biases in risky decision making.

In summary, the results of the parametric modulation analysis support our prediction that risky decision making on behalf of another person may involve additional cognitive processes, including effort-based decision making, self-regulation, and ToM functions. Alternatively, the cognitive/rational system might outweigh the affective/experiential system in risky decision making on behalf of others, given the evidence that links the DMPFC to cognitive processes and the amygdala to affective processes.

This study also unfolds important questions that need to be addressed in future projects. First, we could not determine the relationship between individual differences in brain activity and behavioral responses. We computed a self–other difference index score for each participant and examined the relationship between neural activity and behavioral results. In contrast with our predictions, however, we failed to find statistically significant correlations between them. Although the exact reason for this failure is currently unknown, it may be that few participants showed sufficiently large self–other difference indices. This may have caused limitations in individual variability that obscured the relationship between participants’ decisions and neural responses. Given the role of the prosocial trait in this task, as revealed in the second behavioral study, it would be interesting for a future fMRI study to select participants with a wide range of prosociality. Similarly, it would be interesting to investigate the neural underpinnings of prosocial orientation during self–other decision making, considering our finding from the additional behavioral experiment that increased prosocial orientation reduced self–other differences. We envision future studies to address this important issue.

Second, in this experimental design, we kept the magnitude of gain/loss for each option constant to minimize noise due to variable reward magnitude; we varied only the reward’s attainability (*via* manipulation of winning probability), which modulated the attractiveness of the risky option. Therefore, the difference between the EVs of the high-risk and low-risk options changed with the winning probability, whereas the risk of each option (defined as outcome variance; Markowitz, 1952) remained constant across different levels of winning probability (see Table S2 in Supplementary Material). This feature of our experimental design may leave room for alternative interpretation of the behavioral results. More specifically, the greater sensitivity to winning probability in the decision-for-self condition may simply reflect choices based on the EV of the risky option. Likewise, we cannot completely rule out the possibility that people may have chosen the high-risk option for others less than for themselves out of spite, that is, with the intention of lowering the benefits of others. In this sense, particular caution may be necessary in interpreting the observed correlations between neural activity and the model parameters, and future study should allow for more-systematic manipulation of the EV and risk values for each option.

Third, we did not measure the various psychological factors that could have affected the self–other difference. For instance, it is possible that the subjective social distance between a participant and another person for whom the participant made the decision could have affected his/her decisions, although we explicitly told the participants that they were making decisions for an anonymous person. It would be interesting to test the effect of social distance by comparing decisions made for individuals with whom the subject is close with decisions made for strangers. In addition, we could not confirm which of the psychological processes discussed above is the most prominent driver of the self–other difference. Future studies using different types of tasks or including additional behavioral and physiological measurements, such as eye movements, skin conductance response, or glucose consumption levels, could further elucidate the mechanisms underlying the self–other discrepancy in risky decision making.

The present study included direct comparisons between risky decisions for self and other in a single experiment and provided the first evidence of differences in neural processes between risky financial decisions on behalf of oneself and those on behalf of other. Reward systems were activated when people decided for themselves, whereas the ToM network became more active when subjects made decisions for another person. Most importantly, activity in the neural loci of value computation differed between risky decisions for oneself and for others: the amygdala and DMPFC were associated with decisions on behalf of oneself and others, respectively. Our findings suggest that affective processes have greater weight than cognitive processes in risky decision making for self. On the other hand, decision making for others seems to be a more difficult and effortful process that engages cognitive systems and emotional regulation, in which ToM functions might also participate. We expect future research to follow up on the present findings with the aim of providing a more-complete understanding of the neural mechanisms underlying prosocial and other-regarding behaviors.

## Conflict of Interest Statement

The authors declare that the research was conducted in the absence of any commercial or financial relationships that could be construed as a potential conflict of interest.

## Supplementary Material

The Supplementary Material for this article can be found online at http://www.frontiersin.org/Decision_Neuroscience/10.3389/fnins.2013.00015/abstract
